# Potential Therapeutic Targets and Emerging Strategies to Promote Hematoma Resolution in Intracerebral Hemorrhage

**DOI:** 10.31083/RN46121

**Published:** 2025-12-22

**Authors:** Shuling Wan, Xunming Ji, Ran Meng, Min Li

**Affiliations:** ^1^Department of Neurology, Xuanwu Hospital Capital Medical University, 100053 Beijing, China; ^2^Department of Neurosurgery, Xuanwu Hospital Capital Medical University, 100053 Beijing, China

**Keywords:** intracerebral hemorrhage, hematoma absorption, novel therapy, phagocytosis, glymphatic system, meningeal lymphatic vessels, hemorragia intracerebral, absorción de hematomas, terapia novedosa, fagocitosis, sistema glinfático, vasos linfáticos meníngeos

## Abstract

Intracerebral hemorrhage (ICH) is a devastating stroke subtype with high morbidity and mortality. Beyond primary injury from blood extravasation, secondary injury driven by erythrocyte lysis and its toxic degradation products exacerbates inflammation, oxidative stress, and neuronal damage. Accelerating endogenous hematoma resolution, including the removal of erythrocytes and their byproducts, represents a promising therapeutic strategy. This review systematically delineates three key mechanisms of hematoma resolution post-ICH: (1) erythrophagocytosis by microglia/macrophages through Tyro3, Axl, and Mertk (TAM) receptors, the cluster of differentiation (CD) 36 receptor, the triggering receptor expressed on myeloid cells 2, and the signal regulatory protein α receptor; (2) clearance of hemolytic products through the hemoglobin-haptoglobin-CD163 and hemin-hemopexin-CD91 axes; and (3) glymphatic and meningeal lymphatic drainage. Pharmacological, genetic, and physical interventions targeting these pathways have demonstrated potential to enhance phagocytosis, promote glymphatic and meningeal lymphatic function, accelerate hematoma resolution, and improve neurological outcomes in ICH models. By leveraging the intrinsic clearance mechanisms of the intracerebral hematoma, this review highlights promising therapeutic targets and strategies to overcome current clinical limitations and demonstrates significant translational potential.

## Abstract (es)

La hemorragia intracerebral (ICH, intracerebral hemorrhage) es un subtipo de accidente cerebrovascular devastador con una alta morbilidad y mortalidad. Más allá de la lesión primaria causada por la extravasación de sangre, la lesión secundaria provocada por la lisis de los eritrocitos y sus productos de degradación tóxicos exacerba la inflamación, el estrés oxidativo y el daño neuronal. Por ello, acelerar la resolución endógena del hematoma, que incluye eliminar los eritrocitos y sus subproductos, representa una estrategia terapéutica prometedora. Esta revisión describe sistemáticamente tres mecanismos clave de resolución del hematoma tras una ICH: (1) la eritrofagocitosis por parte de la microglía/macrófagos a través de los receptores TAM (Tyro3, Axl y Mertk), el grupo de diferenciación (CD, cluster of differentiation) 36, el receptor activador expresado en células mieloides 2 y el receptor de la proteína reguladora de señales α; (2) la eliminación de productos hemolíticos a través de los ejes hemoglobina-haptoglobina-CD163 y hemina-hemopexina-CD91; y (3) el drenaje glinfático y linfático meníngeo. Las intervenciones farmacológicas, genéticas y físicas dirigidas a estas vías han demostrado su potencial para mejorar la fagocitosis, favorecer la función glinfática y linfática meníngea, acelerar la resolución del hematoma y mejorar los resultados neurológicos en modelos de ICH. Al hacer uso de los mecanismos de eliminación intrínsecos del hematoma intracerebral, esta revisión destaca objetivos y estrategias terapéuticas prometedoras para superar las limitaciones clínicas actuales y demuestra un importante potencial traslacional.

## 1. Introduction

Intracerebral hemorrhage (ICH), a non-traumatic focal hemorrhage within the brain parenchyma, is the most devastating stroke subtype [1, 2]. Although it represents approximately 20% of all strokes, it accounts for 49.6% of stroke-related disability-adjusted life years [1, 3]. Prognosis of ICH is poor, with a 1-year survival rate of 46%, which declines to 29% at 5 years, and only 33.2% achieving functional independence at 3 months [4, 5].

Brain injury after ICH involves primary and secondary mechanisms. Primary injury arises from tissue damage and mass effect due to blood extravasation. Hematoma expansion, seen in 70% of patients during the acute phase, worsens intracranial pressure and is a strong predictor of poor outcome [6]. Secondary injury is triggered by hemoglobin (Hb) from lysed erythrocytes and its breakdown products (hemin, iron), which drive immune-inflammatory reactions, oxidative stress, blood-brain barrier (BBB) disruption, cerebral edema, and neuronal death [7, 8]. Accelerating hematoma clearance may therefore improve recovery.

Therapeutic options for ICH remain limited to supportive and surgical hematoma evacuation, as emphasized in the latest American Heart Association (AHA)/American Stroke Association (ASA) and European Stroke Organization (ESO) clinical guidelines [9, 10]. In MISTIE III trial, exploratory analysis found better outcomes in patients with post-evacuation hematoma volumes <15 mL [11]. The ENRICH trial showed that minimally invasive surgery within 24 hours improved 180-day outcomes in ICH [12]. However, complications such as rebleeding, tissue injury, infection, and incomplete evacuation have spurred interest in enhancing endogenous hematoma clearance as a potential alternative or adjunctive therapeutic strategy. Moreover, in patient ineligible for surgery, early enhancement of hematoma absorption to alleviate mass effect may promote functional recovery.

This review summarizes therapeutic strategies to enhance endogenous hematoma clearance after ICH, focusing on promoting erythrocyte phagocytosis via the activation of TAM (Tyro3, Axl and Mertk) receptors, cluster of differentiation (CD) 36, and triggering receptor expressed on myeloid cells 2 (TREM2), and the inhibition of signal regulatory protein α (SIRPα)-CD47 pathway; accelerating hemolytic product removal through the Hb-haptoglobin (Hp)-CD163 and hemin-hemopexin (Hx)-CD91 pathways; and enhancing glymphatic and meningeal lymphatic clearance.

## 2. Phagocytosis of Erythrocyte

**Fig. 1.**
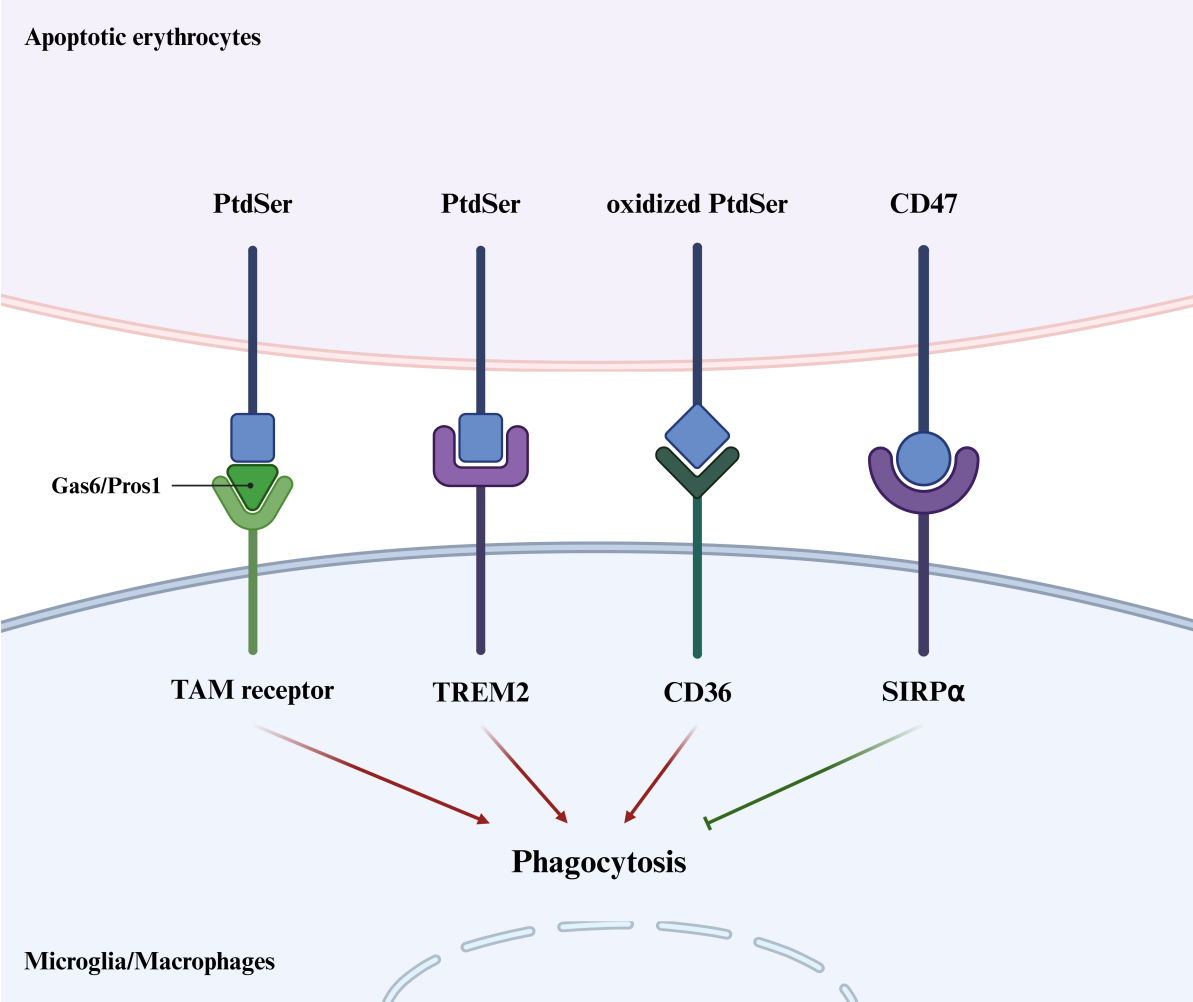
**Erythrophagocytosis of microglia/macrophages**. Microglia/macrophages initiate phagocytosis of apoptotic erythrocytes by recognizing pro-phagocytic signals, phosphatidylserine (PtdSer) or oxidized PtdSer, through TAM (Tyro3, Axl and Mertk) receptors via the bridging ligands growth arrest-specific protein 6 (Gas6)/protein S (Pros1), triggering receptor expressed on myeloid cells 2 (TREM2) and cluster of differentiation (CD) 36. Conversely, CD47 on erythrocytes serves as an anti-phagocytic signal that inhibits phagocytic activity by binding to signal regulatory protein α (SIRPα) on the microglia/macrophages. Created with BioRender.com.

A critical step in hematoma clearance involves the phagocytosis of extravasated erythrocytes by brain-resident microglia and infiltrating macrophages [13]. Erythrocytes exhibit both pro-phagocytic and anti-phagocytic signals on surface. The phagocytic clearance of erythrocytes by microglia/macrophages is regulated through four main pathways: the TAM receptor-growth arrest-specific protein 6 (Gas6)/protein S (Pros1)-phosphatidylserine (PtdSer) pathway, the CD36-oxidized PtdSer pathway, the TREM2-PtdSer pathway, and the SIRPα-CD47 pathway (Fig. 1).

### 2.1 TAM Receptors-Gas6/Pros1-PtdSer Pathway

Following ICH, the hypoxic, oxidative, and pro-inflammatory microenvironment induces erythrocyte apoptosis. PtdSer exposed on the surface of apoptotic erythrocytes acts as a pro-phagocytic signal [14]. The TAM receptor family on microglia/macrophages recognizes PtdSer through the bridging ligands Gas6 and Pros1, thereby triggering apoptotic cell clearance [15]. Post-ICH, Axl and Mertk are upregulated in microglia/macrophages [16, 17, 18]. Axl/Mertk double knockout markedly reduces macrophage erythrophagocytosis in ICH mice, resulting in larger hematomas, greater iron deposition, and worse neurological deficits [18]. Conversely, recombinant Gas6 promotes hematoma resolution, attenuates edema, and improves neurological function via Axl activation [16, 17].

TAM signaling can be inhibited by a disintegrin and metalloproteinase (ADAM)10 and ADAM17, which cleave the extracellular domain of TAM receptors to generate soluble ligand-binding fragments that competitively bind to Gas6 [19, 20]. Inhibition of ADAM10/ADAM17 enhances microglial/macrophage clearance of apoptotic cells [20]. Fan *et al*. [21] developed a pH-responsive neutrophil membrane-based nanoplatform carrying the ADAM17 inhibitor GW280264X and the liver X receptor agonist desmosterol. This platform enables targeted delivery to injury sites, promoting erythrophagocytosis, accelerating hematoma clearance, and improving functional recovery [21]. Taken together, activation of the TAM receptors-Gas6/Pros1-PtdSer pathway may provide a viable therapeutic approach to accelerate hematoma resolution and promote neurological recovery after ICH.

### 2.2 CD36-oxidized PtdSer Pathway

CD36, a class B scavenger receptor, is critical for phagocytosis [22]. Its ectopic expression endows non-phagocytic cells with phagocytic capability, whereas genetic deletion or antibody-mediated blockade significantly impairs phagocyte activity [23, 24, 25]. In phagocytes, CD36 primarily recognizes oxidized PtdSer exposed on apoptotic cells, thereby triggering phagocytosis [26]. Deficiency of CD36 in patients or animal models leads to delayed hematoma clearance and worsened neurological outcomes due to impaired erythrophagocytosis of microglia/macrophages [27].

At transcriptional level, CD36 expression is regulated by peroxisome proliferator-activated receptor γ (PPARγ) and nuclear factor erythroid 2-related factor 2 (Nrf2) [28]. Activation of PPARγ or Nrf2 enhances CD36 expression and erythrophagocytosis of microglia/macrophages, thereby promoting hematoma resolution and neurological recovery after ICH [29, 30, 31]. Pharmacological activation of PPARγ by thiazolidinediones such as pioglitazone and rosiglitazone has demonstrated pro-phagocytic effects in atherosclerosis, underscoring the translational potential of targeting this pathway in ICH models [32]. In 2013, Gonzales *et al*. [33] initiated a randomized controlled trial to investigate the PPARγ agonist pioglitazone for the treatment of spontaneous ICH (NCT00827892), but the final results have yet to be reported. Post-translational modifications, such as SUMOylation, can also enhance microglial CD36 expression and erythrophagocytosis, promote hematoma absorption and alleviate neurological deficits [34].

Moreover, CD36-mediated phagocytosis is modulated by inflammatory signals: pro-inflammatory cytokines such as tumor necrosis factor-α (TNF-α) and interleukin (IL)-1β suppress CD36 expression, delaying hematoma resolution and exacerbating neurological deficits, whereas the anti-inflammatory cytokine IL-10 enhances CD36 expression, accelerating hematoma clearance and functional recovery [27]. Furthermore, the soluble extracellular domain of TREM2 impairs microglial/macrophage erythrophagocytosis by inhibiting vacuolar protein sorting 35-mediated CD36 recycling and promoting lysosomal degradation of non-recycled CD36, ultimately delaying hematoma clearance and worsening neurological deficits [35]. Non-pharmacological interventions, including pulsed electromagnetic field therapy and remote ischemic conditioning (RIC), similarly promote hematoma resolution via CD36 modulation [36, 37]. These findings suggest that activating the CD36-oxidized PtdSer pathway could provide a therapeutic strategy to enhance hematoma resolution after ICH.

### 2.3 TREM2-PtdSer Pathway

TREM2, a type I immunoglobulin superfamily cell-surface receptor comprising a variable immunoglobulin domain, a transmembrane region, and a short cytoplasmic tail, is primarily expressed on microglia, macrophages, and dendritic cells [38]. Similarly, TREM2 is critical for phagocytic function. Transfection of the *TREM2* gene confers phagocytic activity to Chinese hamster ovary cells that natively lack known phagocytic receptors [39]. In addition, TREM2 has been demonstrated to regulate phagocytosis in both microglia and macrophages [39, 40]. The TREM2 receptor on the surface of microglia/macrophages can be activated by binding to PtdSer exposed on apoptotic cells [41]. In ICH models, TREM2 knockout impairs hematoma clearance and worsens neurological deficits, whereas microglia-specific overexpression of TREM2 accelerates hematoma resolution and neurobehavioral recovery [35]. Furthermore, TREM2 agonistic antibody AL002, have been investigated for the treatment of Alzheimer’s disease and have demonstrated favorable safety and tolerability profiles in phase I clinical trials, suggesting that pharmacological modulation of the TREM2-PtdSer pathway could potentially be repurposed to promote hematoma resolution following ICH [42].

### 2.4 SIRPα-CD47 Pathway

CD47, an integrin-associated protein on erythrocytes, functions as an anti-phagocytic signal by binding the inhibitory receptor SIRPα on microglia/macrophages, thereby suppressing phagocytosis [43]. In a porcine ICH model, CD47 expression in white and gray matter increased within 4 hours and remained elevated for up to 14 days [44]. Erythrocytes lacking CD47 are more readily phagocytosed than wild-type cells [45]. Compared with wild-type erythrocytes, nude mice injected with CD47 knockout erythrocytes exhibited faster hematoma resolution, reduced brain edema, and fewer neurological deficits [46]. Similarly, anti-CD47 antibodies enhances hematoma clearance, attenuates brain injury and reduces neurological deficits in ICH models [47, 48, 49, 50]. However, it is particularly noteworthy that CD47 is expressed not only on erythrocytes but also on neurons [44]. Non-specific CD47 blockade may lead to unintended phagocytosis of neurons. Developing strategies to specifically target erythrocyte CD47 may be essential for minimizing off-target effects [51].

Furthermore, strategies that inhibit SIRPα on microglia and macrophages offer an alternative approach to promote hematoma clearance. Yu *et al*. [52] developed a pH-responsive nano-regulator composed of Mg^2+^ and a SIRPα DNAzyme, which releases its components in acidic environments. Mg^2+^ activates the SIRPα DNAzyme, disrupting CD47-SIRPα signaling pathway and thereby enhancing erythrophagocytosis and accelerating hematoma clearance. Collectively, approaches that disrupt CD47-SIRPα pathway represent a promising strategy to facilitate hematoma resolution. Anti-CD47 antibodies such as magrolimab have advanced to phase III clinical trials for myelodysplastic syndrome and acute myeloid leukemia, providing a conceptual framework for application in ICH [53, 54].

## 3. Clearance of Hemolytic Product

**Fig. 2.**
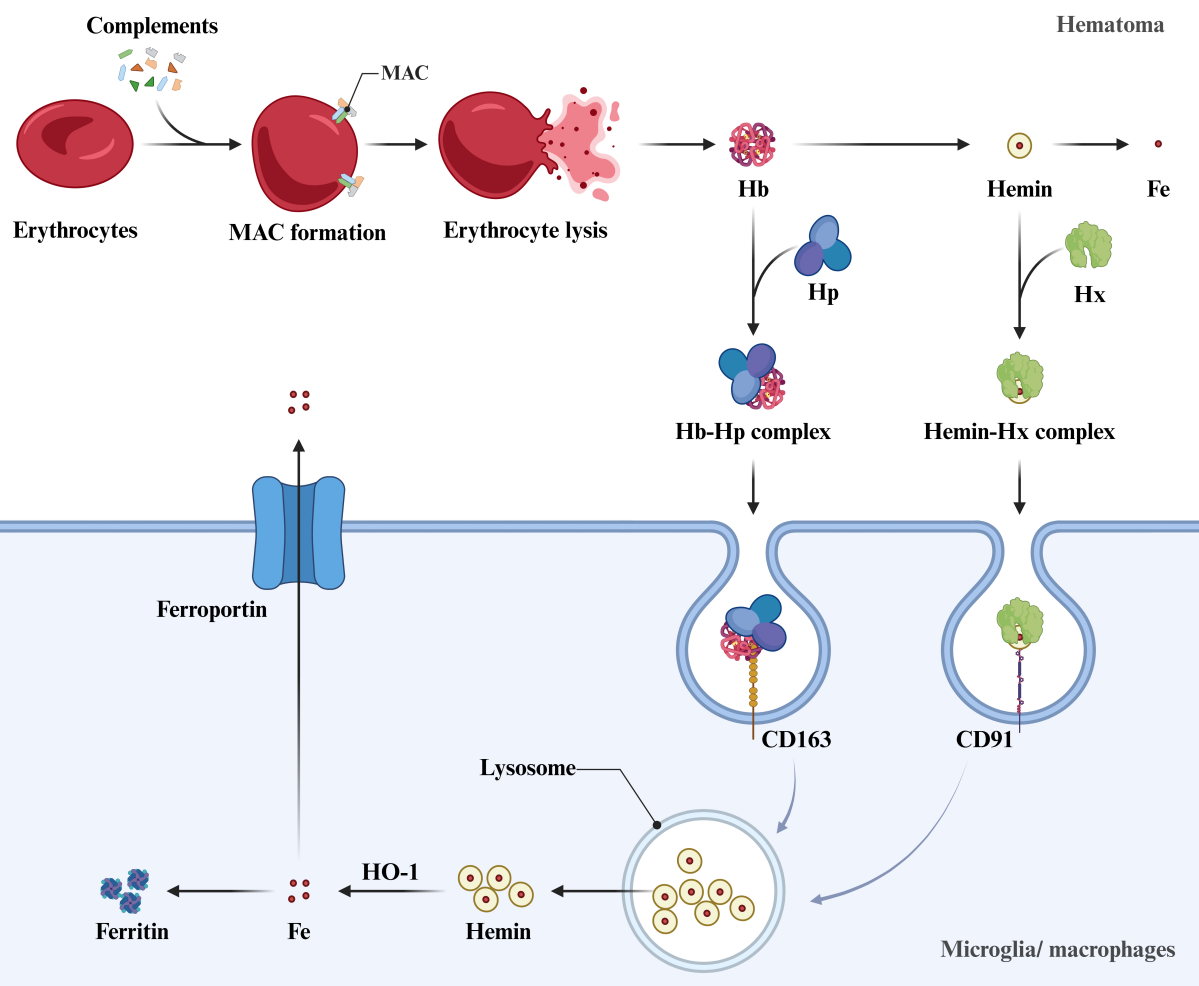
**Complement-mediated erythrocyte lysis and microglial/macrophage clearance of hemolytic products**. Following intracerebral hemorrhage, complement system activation and membrane attack complex (MAC) formation induces erythrocyte lysis within the hematoma, releasing hemoglobin (Hb) which is subsequently metabolized into hemin and iron ions. Microglia/macrophages phagocytose Hb and hemin through the Hb-haptoglobin (Hp)-CD163 pathway and the hemin-hemopexin (Hx)-CD91 pathway. Then, Hb is degraded in lysosomes to release hemin, which is metabolized in the cytosol by heme oxygenase-1 (HO-1) into Fe^2+^, biliverdin, and carbon monoxide. Fe^2+^ is captured by ferritin or transported extracellularly via ferroportin. Created with BioRender.com.

Within 24 hours post-ICH, erythrocytes in the hematoma core undergo complement-mediated lysis and release Hb, which is subsequently degraded into neurotoxic hemin and iron ions [55]. Resident microglia and infiltrating macrophages uptake free Hb and hemin through the Hb-haptoglobin (Hp)-CD163 and hemin-hemopexin (Hx)-CD91 pathways [56, 57]. After internalization, Hb is degraded in lysosomes to release hemin, which is subsequently metabolized into Fe^2+^, biliverdin, and carbon monoxide by heme oxygenase (HO)-1 in the cytosol. The Fe^2+^ is then captured by ferritin and stored as Fe^3+^, or transported extracellularly via ferroportin [58] (Fig. 2).

### 3.1 Erythrocyte Lysis

As the main cellular component of hematoma after ICH, erythrocytes begin to lysis within 24 hours [55]. The complement cascade plays a crucial role, activated via classical, alternative, and lectin pathways, all converging to generate C3 convertase [59]. RNA sequencing shows increased expression of classical and alternative pathway components post-ICH, while lectin pathway changes are minimal [60].

C3 is cleaved into C3a and C3b, leading to C5 convertase formation, cleavage of C5, and subsequent assembly of membrane attack complex (MAC, C5b-C9) that disrupts cell membranes and induces lysis [59]. RNA sequencing shows that *C3* mRNA is primarily expressed in microglia. A study incorporating both clinical and murine data demonstrates that plasma C3 levels are elevated following ICH and correlate with hematoma volume and disease severity [61]. C3-deficient mice exhibit reduced erythrocyte lysis, less brain injury, and improved neurological recovery, indicating that targeting C3 may mitigate erythrocyte lysis post-ICH [62]. CR2-Crry, a recombinant fusion protein inhibiting C3 activation, offers neuroprotection in murine hemorrhagic models [63]. Additionally, normobaric hyperoxia has been shown to reduce C3 levels, promote neurological recovery, and enhance hematoma resolution following ICH [61].

MAC, the terminal product of the complement cascade, directly mediates erythrocyte lysis and Hb release [64]. Animal studies demonstrate that MAC accumulates within hematomas and colocalizes with erythrocytes [63, 65]. Inhibitors such as N-acetylheparin and aurintricarboxylic acid block MAC formation, thereby reducing erythrocyte lysis and brain injury in models of ICH [64]. Depletion of the gut microbiota upregulates regulatory T cells, which in turn reduces MAC formation, facilitates neurological recovery, and accelerates hematoma resolution [66].

### 3.2 Hb Clearance

Hb and its degradation products induce neurotoxicity via multiple pathways, making extracellular Hb clearance a key therapeutic target after ICH. The Hb-Hp-CD163 axis is the primary pathway for this process. Hp is an acute-phase α_2_-glycoprotein that binds free Hb with exceptionally high affinity to form Hb–Hp complexes. These complexes inhibit Hb-induced oxidative damage and facilitate Hb clearance via the CD163-mediated endocytic pathway [67, 68]. Following ICH, oligodendrocytes can synthesize and secrete Hp to promote Hb clearance [69]. The neuroprotective effects of Hp in both *in vivo* and *in vitro* ICH models are highly complex. They appear to depend on factors such as age, disease stage, and the local microenvironment [69, 70, 71].

CD163 is a scavenger receptor highly expressed on microglia/macrophages. It mediates the endocytosis of Hb-Hp complexes and free Hb in Hp deficiency [56]. CD163-positive microglia/macrophages accumulate in perihematomal regions after ICH [65]. Studies indicate that upregulating CD163 expression in microglia/macrophages after ICH through activation of the PPAR-γ pathway (via PPAR-γ agonist monascin and chemokine fractalkine) or the Nrf2 pathway (via C-C motif chemokine ligand 17) promotes hematoma clearance [72, 73, 74]. However, Leclerc *et al*. [75] revealed a biphasic role of CD163 deficiency in ICH, with early protective effects shifting to later detrimental consequences.

Notably, neuronal CD163 expression is also upregulated following ICH or Hb stimulation [70, 76]. However, neurons express very low levels of ferritin, which limits the degradation of heme products and ultimately leads to neuronal injury or death [70]. Selectively upregulating CD163 expression in microglia/macrophages while suppressing its expression in neurons may be a potential therapeutic strategy to mitigate brain injury following ICH. 5α-androst-3β,5α,6β-triol (TRIOL), selectively increases CD163 expression in microglia/macrophages without affecting neuronal CD163 levels [77]. Moreover, deferoxamine attenuates ICH- and Hb-induced neuronal CD163 upregulation and associated neuronal damage both *in vitro* and *in vivo* [76].

### 3.3 Hemin Clearance 

Following ICH, the released Hb is subsequently degraded to hemin, which contributes to oxidative stress, inflammation, and neuronal injury [78]. In porcine autologous blood injection models, hemin levels in the hematoma and perihematomal tissue rise sharply within 24 hours, peak at day 3, and remain elevated through day 7, exceeding *in vitro* thresholds for neuronal death [79]. The Hb-Hx-CD91 axis is the primary pathway for hemin clearance.

Hx is a 60 kDa glycoprotein normally present at very low levels in the brain. After ICH, the level of Hx in the brain increases markedly due to the entry of peripheral Hx into the brain tissue through the hemorrhage or the disrupted blood-brain barrier, as well as increased local secretion of Hx [80]. Hx binds free heme with high affinity, and the resulting complex can be endocytosed via CD91/low-density lipoprotein receptor-related protein 1 [57]. Perihematomal microglia/macrophages upregulate both Hx and CD91 to facilitate hemin clearance [79, 81]. Microglia/macrophage-specific CD91 knockout impairs hematoma resolution, exacerbates oxidative stress, and worsens neurological deficits following ICH [81]. Therapeutically, cerebral Hx overexpression via recombinant adeno-associated virus vectors reduces lesion volume and alleviates neurological deficits in ICH models. However, systemic administration of exogenous Hx fails to improve neurological function [80, 82]. Administration of exogenous CD91 promotes hemin clearance, reduces oxidative stress and neuronal damage, and markedly decreases hematoma volume and neurological deficits, while these neuroprotective effects are partially reversed by CD91 siRNA. Additionally, rosuvastatin upregulates CD91 expression and diminishes neuropathological damage in ICH mice [83].

Hemin is degraded into Fe^2+^ by HO in the cytosol [58]. Among the three isoforms (HO-1, HO-2, and HO-3), HO-1 and HO-2 play major roles in hemin degradation after ICH [77, 84]. Increased expression of HO-1 in microglia/macrophages and HO-2 in neurons is observed in the perihematomal region [77, 84]. The role of HO-1 appears to be phase-dependent. Pharmacologically induced HO-1 upregulation exacerbates brain injury in the early phase (day 1–3) of ICH but promotes hematoma resolution and neurological recovery in the late phase (day 28) [85, 86]. The optimal timing and extent of HO-1 activation required for therapeutic benefit remain to be further elucidated. Astrocytic HO-1 overexpression reduces cell death, BBB disruption, and neurological deficits in ICH models [87, 88]. The role of HO-2 remains controversial. *In vitro* studies by Rogers *et al*. [89] and Regan *et al*. [90] found that HO-2 knockout reduces neuronal sensitivity to Hb and hemin toxicity, whereas Wang *et al*. [91] reported opposite conclusions. *In vivo* studies of HO-2 knockout exhibited significant model-dependent variations [91, 92, 93, 94, 95, 96, 97].

## 4. Glymphatic and Meningeal Lymphatic Clearance

### 4.1 Glymphatic Clearance

**Fig. 3.**
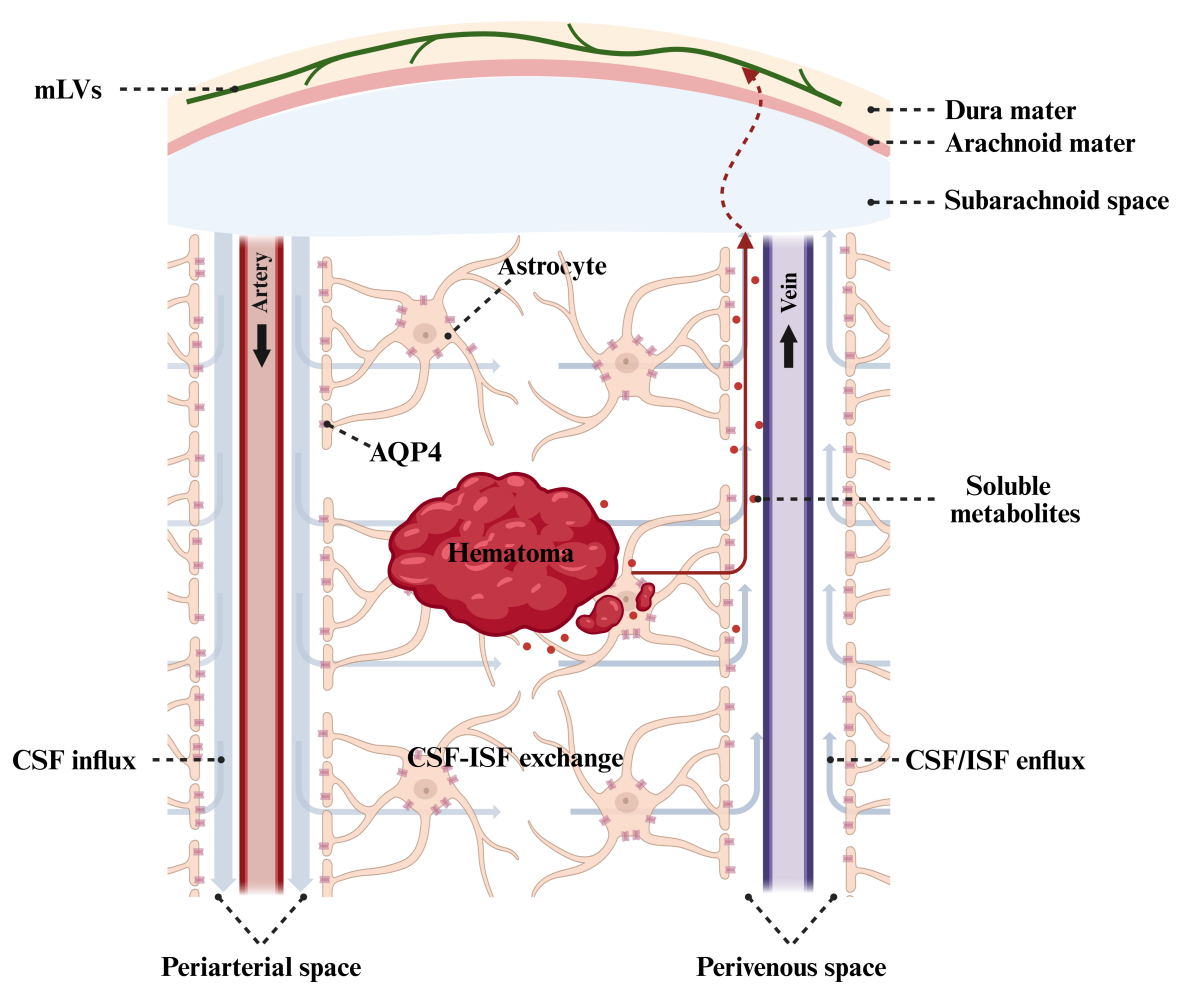
**Endogenous hematoma clearance mediated by the glymphatic system and meningeal lymphatic vessels**. Cerebrospinal fluid (CSF) in the subarachnoid space flows into the periarterial spaces, enters the interstitial space via aquaporin-4 (AQP4) channels on astrocytic endfeet, mixes with interstitial fluid (ISF) containing soluble hematoma metabolites, subsequently refluxes along the perivenous spaces back to the subarachnoid space, and ultimately drains to the cervical lymph nodes via meningeal lymphatic vessels (mLVs), thereby promoting hematoma clearance. Created with BioRender.com.

The glymphatic system is a perivascular network formed by astrocytic endfeet [98]. Cerebrospinal fluid (CSF) in the subarachnoid space flows along the periarterial spaces, then enters the brain parenchyma through aquaporin-4 (AQP4) channels on astrocytic endfeet. Here it exchanges with interstitial fluid, subsequently drains along perivenous spaces back to the subarachnoid space, and is ultimately transported to cervical lymph nodes (CLNs) via meningeal lymphatic vessels (mLVs), thereby achieving the clearance of metabolic waste from the parenchyma (Fig. 3) [99, 100]. An experimental study indicates that the AQP4-dependent glymphatic pathway contributes to hematoma clearance. The AQP4 activator mifepristone enhances AQP4 expression and polarization after ICH, improves glymphatic function and lymphatic drainage, thereby accelerating hematoma clearance, reducing neuronal injury, and improving neurological outcomes. Conversely, AQP4 inhibition or knockout produced opposite effects [101]. Melatonin treatment similarly improved AQP4 polarization, glymphatic function, lymphatic drainage, and hematoma resolution, while its effects were blocked by the receptor antagonist luzindole [98]. Collectively, these findings highlight the therapeutic potential of targeting the AQP4-glymphatic pathway in ICH.

Various drugs and interventions, including the transient receptor potential vanilloid 4 inhibitor HC-067047, β-hydroxybutyrate, nimodipine, oxytocin, fingolimod, IL-33, the small molecule OAB-14, the matrix metalloproteinase-9 inhibitor GM6001, traditional Chinese medicines Xuefu Zhuyu Decoction and Yuanzhi Powder, RIC, very low-intensity ultrasound, and repetitive transcranial magnetic stimulation (rTMS) have been shown to improve glymphatic function in different disease models [102, 103, 104, 105, 106, 107, 108, 109, 110, 111, 112, 113, 114]. However, their efficacy in ICH models has not yet been verified and requires further investigation.

### 4.2 Meningeal Lymphatic Clearance

Current studies indicate that erythrocytes and solutes in the brain parenchyma can be drained to CLNs via mLVs after ICH (Fig. 3) [115, 116]. During the acute phase of ICH (within 3 days), mLV function is impaired, whereas during the recovery phase (days 10–14), lymphatic drainage and lymphangiogenesis are enhanced and can persist for months [115, 117]. In ICH models, cilostazol, panax notoginseng saponins and simvastatin enhance meningeal lymphatic function, promotes erythrocyte drainage to CLNs, enhances hematoma clearance, reduces neuronal damage, and improves neurological function [115, 118, 119]. As these medications may increase the risk of bleeding, their use should be exercised with caution. Conversely, mLV ablation or functional inhibition impairs hematoma clearance [115]. Overall, enhancing meningeal lymphatic clearance represents a promising strategy for hematoma resolution and functional recovery after ICH.

Vascular endothelial growth factor-C, ketoprofen, 9-cis retinoic acid, Yoda1, down syndrome critical region 1, transcranial photobiomodulation, and rTMS have also been shown to promote meningeal lymphatic drainage [114, 120, 121, 122, 123]; however, their efficacy in ICH remains unverified, and further studies are needed to clarify clinical applicability.

## 5. Discussion

Although erythrophagocytosis, hemolytic product clearance, and lymphatic drainage have been described as distinct mechanisms, they operate in a tightly coordinated and temporally ordered manner during hematoma resolution. In the acute phase, microglial and macrophage erythrophagocytosis initiates the removal of intact erythrocytes, thereby limiting erythrolysis and the release of toxic hemolytic products. Subsequently, the Hb-Hp-CD163 and Hemin-Hx-CD91 pathways become predominant, facilitating detoxification and iron sequestration within phagocytes. As debris and soluble metabolites accumulate, the glymphatic and meningeal lymphatic systems gradually assume a dominant role, draining residual byproducts and inflammatory mediators from the parenchyma to peripheral lymph nodes. Spatially, these processes are interlinked, as phagocytes near the hematoma core mediate local degradation, while the glymphatic and meningeal lymphatic networks provide distal clearance routes.

Although numerous preclinical studies have demonstrated the efficacy of interventions that enhance hematoma clearance, translation to clinical application remains challenging. Interspecies differences in immune responses, erythrophagocytic capacity, and glymphatic-lymphatic anatomy limit the extrapolation of animal data to humans. Rodent models often exhibit faster hematoma resolution and milder inflammation than observed clinically, highlighting the need for large-animal or humanized models to validate therapeutic mechanisms [124].

Therapeutic timing is a critical factor. Interventions targeting HO-1 show phase-dependent effects, being detrimental in the early phase but neuroprotective in the late phase. Similarly, the functional capacity of meningeal lymphatic vessels (mLVs) changes over time after hemorrhage, affecting hematoma clearance efficiency. Understanding the temporal dynamics of both HO-1 regulation and mLV function is therefore essential for optimizing therapeutic strategies, as appropriately timed interventions may enhance hematoma resolution while minimizing adverse effects.

The safety of immune modulation therapies also warrants careful consideration. Enhancing microglial or macrophage phagocytosis can accelerate hematoma absorption, but excessive activation may induce secondary inflammation [125]. Likewise, approaches such as CD47 blockade, PPARγ agonists, or HO-1 inducers require precise dose titration to balance efficacy and safety, as off-target effects may further complicate therapeutic outcomes. Emerging nanotherapeutic platforms offer improved brain targeting and sustained drug release, although their biocompatibility, clearance, and long-term toxicity remain insufficiently characterized [126, 127]. Therefore, comprehensive pharmacokinetic and biosafety evaluations are essential before clinical translation.

Finally, integrating these biological insights into the current clinical framework according to the AHA/ASA 2022 and ESO 2023 guidelines will be crucial to achieve therapeutic synergy. The clearance-promoting strategies discussed herein may complement guideline-based management to enhance neurological recovery after ICH.

## 6. Conclusions

Endogenous hematoma resolution after ICH involves coordinated processes of erythrophagocytosis, clearance of hemolytic products, and glymphatic-meningeal lymphatic drainage. Enhancing microglial/macrophage erythrophagocytosis through activation of TAM receptors, CD36, and TREM2 or inhibition of the SIRPα-CD47 pathway plays a central role in promoting hematoma clearance and mitigating secondary brain injury. Inhibiting erythrocyte lysis while facilitating the clearance of hemolytic products via the Hb-Hp-CD163 and Hemin-Hx-CD91 pathways constitutes another critical mechanism. In parallel, enhancement of glymphatic and meningeal lymphatic drainage contributes to the efficient removal of erythrocytes and hemolytic products. Further research is needed to determine the optimal timing, efficacy, and safety of interventions targeting these pathways for potential clinical application.
